# *Mycobacterium abscessus* infection after injection of lipolytic enzymes into abdominal fat

**DOI:** 10.1590/0037-8682-0284-2023

**Published:** 2023-09-22

**Authors:** Marilda Aparecida Milanez Morgado de Abreu, Germana Fernanda de Souza, Marcelo Guimarães Tiezzi, Mariana Baptista Angeluci

**Affiliations:** 1 Universidade do Oeste Paulista, Presidente Prudente, SP, Brasil.; 2 Hospital Regional, Departamento de Dermatologia, Presidente Prudente, SP, Brasil.; 3 Hospital Regional, Departamento de Patologia, Presidente Prudente, SP, Brasil.

**FIGURE 1:**
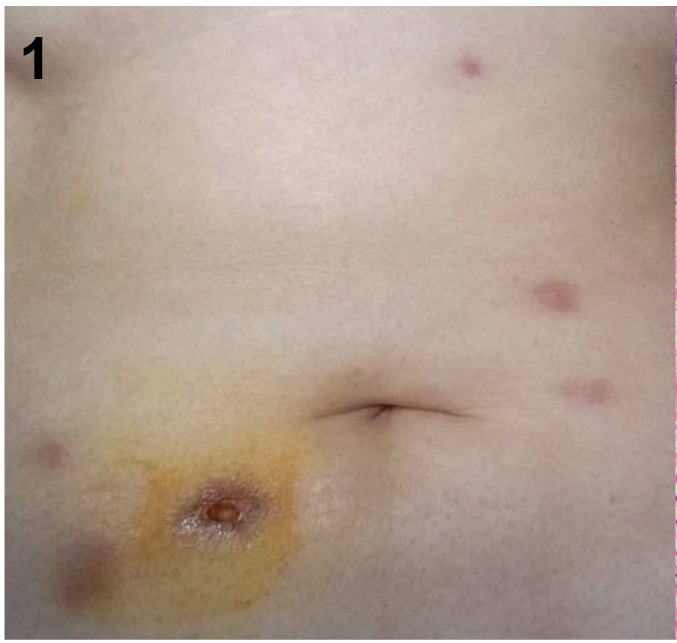
Multiple erythematous nodules on the abdomen, one of them with ulceration.

**FIGURE 2:**
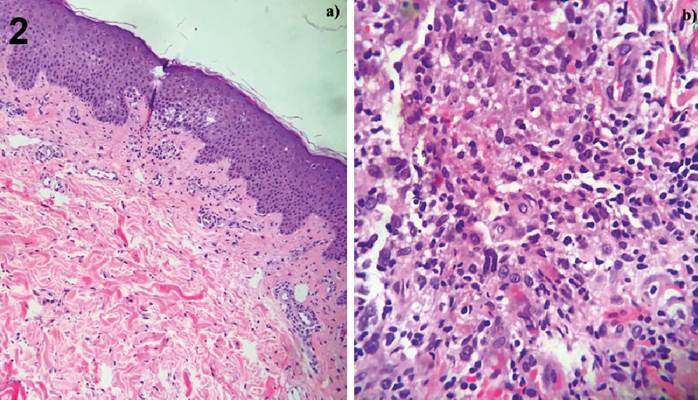
a) Histopathology of biopsy showing acanthosis and lymphoplasmacytic infiltrate in the dermis, outlining granulomas (hematoxylin-eosin ×100). b) Detail of the inflammatory infiltrate (hematoxylin-eosin, ×400).

**FIGURE 3:**
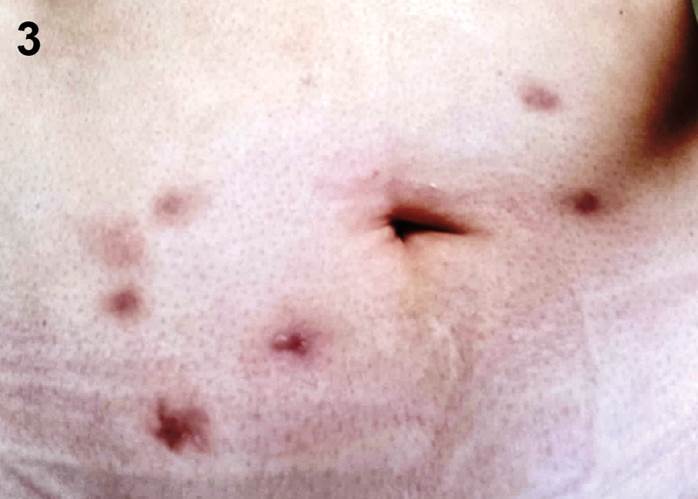
Hyperchromic and atrophic scars after treatment.

The *Mycobacterium abscessus* complex, a part of the nontuberculous mycobacteria (NTM) group, comprises three subspecies: *M. abscessus* subsp. *abscessus*, *M. abscessus* subsp. *massiliense*, and *M. abscessus* subsp. *boletii* ^1^ ^,^ ^2^. These microorganisms are known to cause skin and other soft tissue infections following injuries with nonsterile materials^3^ ^-^ ^5^. This study was approved by the Institutional Ethics Committee. A 38-year-old woman presented with painful inflammatory nodules 2 months after receiving injections of lipolytic enzymes into her abdominal fat (Figure 1). The nodules were tender, and the patient reported pus drainage. Despite a 7-day course of cephalexin and moxifloxacin, her condition did not improve. Azithromycin 500 mg and ciprofloxacin 500 mg daily was initiated. The direct acid-fast bacilli (AFB) examination yielded negative, and the histopathological examination revealed deep granulomatous chronic dermatitis with granulation tissue, without evidence of AFB or fungi (Figure 2). However, a culture of the fragment in Lowenstein-Jensen medium and Mycobacteria Growth Indicator Tube broth with automated fluorimetry detected mycobacteria. *M. abscessus* subsp. *abscessus* was identified through partial sequencing of the rpoB gene and further analyzed by the GenBank international database. After maintaining the azithromycin for 6 months, the lesions resolved leaving scars (Figure 3). The growing interest in aesthetic procedures has led to a significant increase in NTM infections, making them an emerging public health issue. Such procedures should be performed by qualified medical professionals using aseptic techniques and materials. Public health strategies are needed to prevent morbidity and mortality, and it is crucial to educate the public about these risks.
